# Influence of Ionizing Radiation on Two Generations of Cochlear Implants

**DOI:** 10.1155/2015/609607

**Published:** 2015-09-28

**Authors:** Nicolas Guevara, Anaïs Gérard, Jeanne Dupré, Delphine Goursonnet, Michel Hoen, Dan Gnansia, Gaëlle Angellier, Juliette Thariat

**Affiliations:** ^1^Department of Head and Neck Surgery, CHU Nice, 06100 Nice, France; ^2^Department of Radiation Oncology/IBDC CNRS UMR 6543, Cancer Center Antoine-Lacassagne, University Nice Sophia-Antipolis, 06189 Nice, France; ^3^Clinical and Scientific Research Department, Oticon Medical-Neurelec, 06220 Vallauris, France

## Abstract

The purpose of the present study was to test the behavior of two different generations of cochlear implant systems subjected to a clinical radiotherapy scheme and to determine the maximal acceptable cumulative radiation levels at which the devices show out-of-specification behaviors. Using stereotactic irradiation (Cyberknife, 6 MV photon beam), three Digisonic SP and three Neuro devices were submitted to 5 Gy doses that cumulated to 60 Gy (12 sessions) and 80 Gy (16 sessions), respectively. A follow-up series of irradiation was then applied, in which Digisonic SP devices received two additional fractions of 50 Gy each, cumulating to 160 Gy, and Neuro devices three additional fractions of 20, 40, and 150 Gy, cumulating to 290 Gy. Output current values were monitored during the treatment. At clinical doses, with 60 or 80 Gy cumulative radiation exposure, no single measurement showed more than 10% divergence from the reference measure. The cochlear implants tested in this study showed high resistance to clinically relevant cumulative radiation doses and showed no out-of-bounds behavior up to cumulative doses of 140 or 160 Gy. These observations suggest that cochlear implant users can undergo radiotherapy up to cumulative doses well above those currently used in clinical situations without risk of failure.

## 1. Introduction

Cochlear implants (CIs) currently constitute the most successful machine-brain interface in use. Cochlear implantation has changed the lives of numerous patients with severe-to-profound hearing loss. Given the growing number of CI users globally and the relatively high incidence of central nervous system and head and neck radiotherapies [1], the probability of a CI user undergoing radiotherapy has increased to nonnegligible levels, making it important that modern CI devices can undergo such clinical treatments while still operating within their specified range.

**Figure 1:**
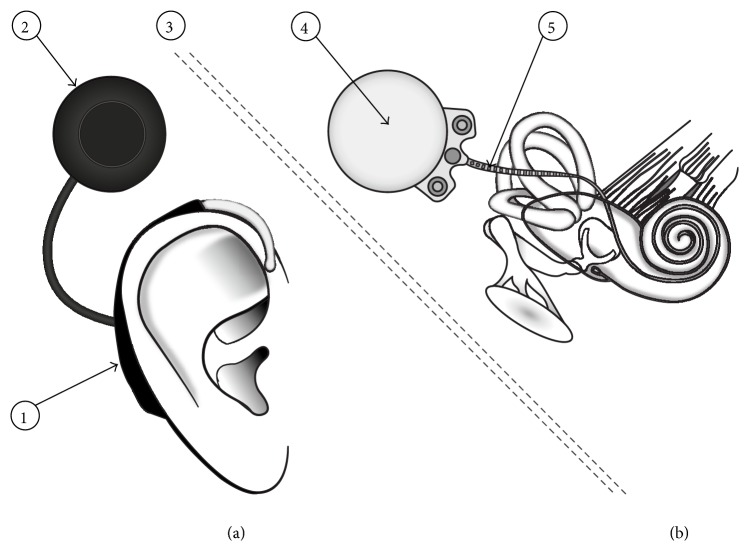
General presentation of the cochlear implant system. (a) The external speech-processor. (b) The implanted receiver/transducer. ① BTE-housed speech processor, ② antenna, ③ skin barrier, ④ body of the receiver, and ⑤ multielectrode array.

The general principle of deafness treatment using CI is to replace absent or damaged components of the ear with an implanted multielectrode array that directly delivers electrical stimulations to the auditory nerve. As illustrated in Figure 1, a CI system is composed of two main parts. The first is an external, removable speech processor (Figure 1(a)). It contains microphones, a digital processor, and batteries. Its function is to capture sound and to process it in order to maximize signal quality and speech intelligibility.

Processed signals and electric power are transmitted to the implanted part of the system, the receiver/stimulator (RS) (Figure 1(b)). The body of the receiver (④ in Figure 1(b)) includes a magnet, which maintains the position of the external antenna and allows signal transmission. It also contains a transducer that interprets instructions coming from the sound processor and translates them into electric pulse trains that are delivered to the auditory nerve via a multielectrode array (⑤ in Figure 1). The array is surgically positioned, via a cochleostomy through the promontory, anteroinferior to the round window. Its final location is inside the* scala tympani* of the cochlea, adjacent to the auditory nerve. CIs are thus sophisticated implantable medical devices that may be affected by radiation, as already observed in implantable cardiac devices [2, 3]. More specifically, ionizing radiation used for radiotherapy may have direct deleterious effects on implant function by influencing the performance of the complementary metal oxide semiconductor (CMOS) components incorporated into the implant [4].

Nevertheless, the risk of implant failure seems to be low, as shown in previous studies suggesting that devices still operate within their specification ranges after relatively high doses of irradiation. Former work measured either radiofrequency- (RF-) lock, that is, the efficiency of data transmission between the external speech processor and the implanted receiver of the CI, or current output. These studies generally reported few failures due to irradiation, with loss of RF-lock, that is, interrupted transmission of information between the speech processor and the receiver, was observed gradually between 50 and 150 Gy [5, 6] while substantial output current modifications were either not observed at the tested radiation levels, for example, 42.5 Gy [7], or observed only with relatively high amounts of cumulative radiation, such as 111 Gy [8] or 150 Gy [6]. These data indicated that radiotherapy could be employed with the external part of the CI system in place. However, the implanted component cannot be removed temporarily, and breakage of the device would require a new surgery with renewed surgical risks for the patient. With the miniaturization of devices, new electronic materials are used, potentially raising the susceptibility of CI devices to radiation because of the increasing number and the specific nature of CMOS components employed [9]. Thus, we tested the resistance of two different generations of internal RS and electrode arrays to radiation, using a linear accelerator delivering 6 MV photon beams (CyberKnife, Accuray, Sunnyvale, California, United States). The Digisonic SP is the current version of an implanted receiver developed by Oticon Medical-Neurelec (Vallauris, France) [10–12]. A new CI system (Neuro) is under development, with an updated mechanical design and electronic components. The goal of the current study was first to evaluate the effects of radiation on these two CI models using radiation schemes comparable to those used in standard radiotherapy protocols. Second, we sought to identify the maximum dose that allowed both CI systems to function within their specification ranges.

## 2. Materials and Methods

### 2.1. Devices

#### 2.1.1. Digisonic SP CI

**Figure 2:**
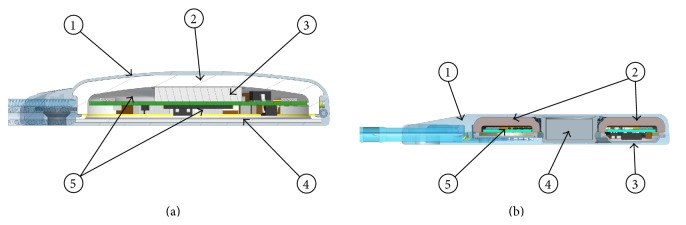
(a) Left: cross section of the* Digisonic SP* cochlear implant. ① Silicone overmolding. ② Ceramic, thickness 1.2 mm. ③ Samarium cobalt magnet, 1.5 mm. ④ Titanium base, 0.4 mm. ⑤ Electronic board: epoxy resin FR-4, 0.4 mm. (b) Right: cross section of the Neuro cochlear implant. ① Silicone overmolding, min. thickness 0.4 mm. ② Ceramic, 1.25 mm: Zircon. ③ Titanium cover, 0.25 mm. ④ Magnet, 3 mm. ⑤ Electronic board and components.

The implant body of the Digisonic SP is a ceramic box sealed with a titanium base (see Figure 2(a)). The RS has a diameter of 30.2 mm and a thickness that varies from 5.75 mm in the center to 4.9 mm at the edges. It is a compact monoblock structure molded in a 0.3 to 0.75 mm thick silicone membrane that contains all of the electronic components, the magnet and coil, and a flat titanium base. The electrodes are made of platinum-iridium, and the electrode array (EA) includes 20 electrodes on a 25 mm active length. The RS is secured to the skull by autotapping titanium screws, through a minimally invasive retroauricular skin incision; the ceramic box is positioned on the temporal bone, and the EA is inserted into the cochlea [12].

#### 2.1.2. Neuro CI

The implant body of the new generation of CI, the Neuro CI (Figure 2(b)), is a donut-shaped monoblock structure composed of one shell of ceramic and one shell of titanium molded in a silicone membrane of 0.4 mm thickness. The RS has a diameter of 30.5 mm and a maximum thickness of 4.5 mm. This structure contains all of the electronic components and allows the insertion of a magnet in the center of the donut shape. The electrodes are made of platinum-iridium, and the electrode array includes 20 electrodes on a 25 mm active length. The EA is connected to the RS with an extensive junction. The RS is secured to the skull by autotapping titanium screws, through a minimally invasive retroauricular skin incision; the donut-shaped box is positioned on the temporal bone, and the EA is inserted into the cochlea.

### 2.2. Device Preparation

**Figure 3:**
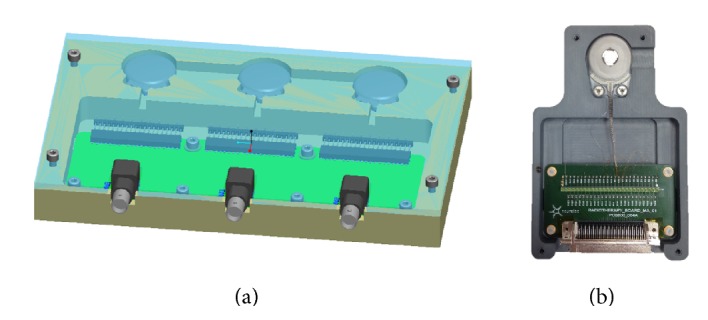
Holders used to present the devices for irradiation. (a) The single box was used for presenting the three Digisonic SP devices and (b) an individual holder, shown without the 5 mm PC covers, was used to hold the Neuro devices.

Three units of each model (Digisonic SP and Neuro) were included in the present irradiation protocol. The devices did not have any electrode array, but the output wires of the receiver were connected to a dedicated board with a 1 kOhm resistance. The implant setup is shown in Figure 3. For the Digisonic SP measurements, all three implants were placed on the same measuring board (Figure 3(a)). The board was constructed from a 30 × 15 cm piece of plastic (PVC) that was molded to hold the implant; it was covered with a 5 mm thick transparent polycarbonate (PC) cover plate to mimic the skin. For the Neuro CI measurements, each implant was placed on a separate board (Figure 3(b)). The boards were 10 × 5 cm pieces of PVC that represented the bones to which implants were screwed using their typical fixation method; they were covered with 5 mm thick sheets of transparent PC to mimic the skin.

### 2.3. Irradiation Schemes

Irradiation was conducted at the Antoine Lacassagne center for proton-therapy (Nice, France), via stereotactic irradiation using a linear accelerator delivering 6 MV photon beams. Setup was calibrated for a dose delivered at 80 cm. All of the implants were first subjected to a fractionated irradiation scheme, receiving a single daily dose of 5 Gy, four days per week, up to a total of 12 sessions (total dose = 60 Gy) for Digisonic SP implants and 16 sessions (total dose = 80 Gy) for Neuro CI devices. Following this first radiotherapeutic protocol, a follow-up series of irradiation was applied to devices still working in their specified range to establish maximal doses before breakdown of the system outside the clinical range. Digisonic SP devices received two additional doses of 50 Gy each over a two-week period, bringing the cumulative irradiation dose to 160 Gy. Neuro CI devices received three supplementary doses of 20, 40, and 150 Gy delivered over three consecutive days, bringing the total irradiation dose at the end of the trial to 290 Gy.

### 2.4. Measurements

To obtain measures in clinically comparable conditions, between each radiation exposure the individual implants were placed in a transportable incubator at 37°C, and speech processors transmitting power and random stimulation patterns were connected to the implants to mimic the function of an implant worn and used by a patient engaged in radiotherapy. After each radiation dose delivery session, output currents were measured at each of the 20 electrode ports. Before the start of the irradiation protocol, reference measures were obtained for each individual device under the same conditions. Devices were considered functional if the output current did not diverge more than 10% from the pretreatment reference measure. This measure is the current benchmark in pacemakers [9] or CI current outputs [7, 8], corresponding to the operating range of devices as guaranteed by manufacturers. Data were expressed as a percentage of the reference measure obtained for each individual device at each individual electrode.

## 3. Results and Discussion

### 3.1. Clinical Fractionating Scheme

#### 3.1.1. Digisonic SP Devices

**Figure 4:**
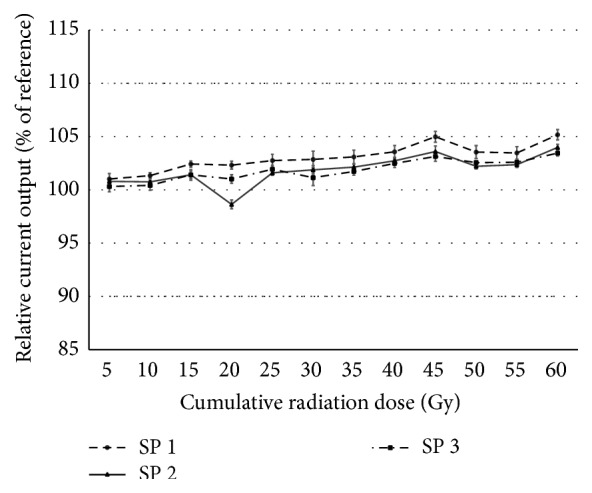
Evolution of output currents for the 3 Digisonic SP devices. Average values across the 20 electrodes over the 12 radiation sessions were expressed in % of reference measure. Dotted lines show the reliability interval of ±10%. Close-up of the 85 to 115% range, error bars show the standard deviation for each measure.

When subjected to 5 Gy once each day on four days per week for up to a total of 12 sessions (CyberKnife, Accuray, Sunnyvale, California, United States, total dose = 60 Gy), none of the 20 electrodes in each of the three tested Digisonic SP devices showed output currents that were out of range (±10% from reference). Because each of the 20 electrodes showed very similar behavior, we averaged the current measures across the electrodes for each tested device.  Figure 4 shows the evolution of the averaged output current over the 12 radiation sessions.

On average, the output currents showed a small but progressive increase over the duration of the irradiation sessions, with maximal values observed at the 12th session, which corresponds to a cumulated radiation dose of 60 Gy. The maximal increases were +5.17%, +3.99%, and +3.45% for Digisonic devices SP1, SP2, and SP3, respectively. All of the measurements remained well within the defined 10% acceptable limit and showed relatively low standard deviations among the electrodes.

#### 3.1.2. Neuro CI Devices

**Figure 5:**
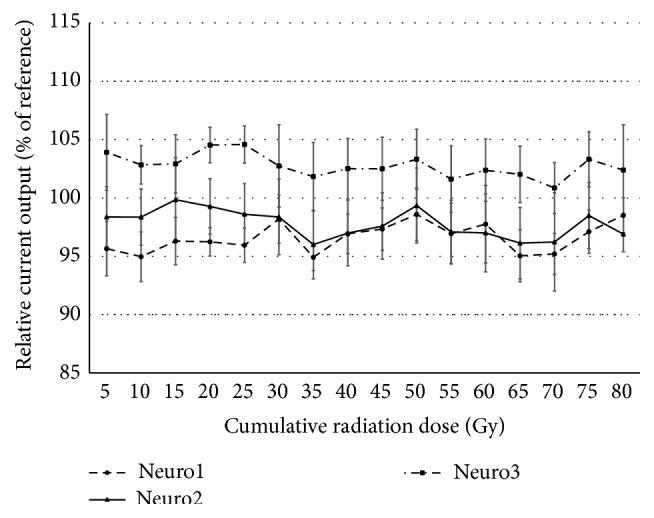
Evolution of output currents for the 3 Neuro CI devices. Average values across the 20 electrodes over the 12 radiation sessions were expressed in % of reference measure. Dotted lines show the reliability interval of ±10%. Close-up of the 85 to 115% range, error bars show the standard deviation for each measure.

The Neuro devices underwent clinical fractionated radiotherapy with a scheme of 5 Gy each day on four days per week, up to a total of 16 sessions (6 MV photon beam, total dose = 80 Gy). During the exposure period, the average current output measurements from the Neuro devices showed small, nonlinear variations (Figure 5). None of the 20 electrodes in any of the three tested devices showed output currents that were out of range (±10% from the reference).

On average, the output currents of the three Neuro devices showed varying behavior with increasing cumulative radiation doses. Two devices (Neuro1 and Neuro2 in Figure 5) showed a small but progressive diminution of output currents across sessions, with final values at the 16th session (corresponding to a cumulative radiation dose of 80 Gy) of −1.49% and −3.10%, respectively. During the irradiation period, the maximal 20-electrode average deviations of Neuro1 and Neuro2 were both observed after treatment session number 7 (35 Gy total) and reached −5.08% and −4%, respectively. The third device (Neuro3) showed a slight increase in average output current, ending at session 16 (80 Gy total) with a variation of +2.39% and showing a maximal variation of +4.58% at session 5 (25 Gy). All of the measurements remained well within the defined ±10% acceptable range despite displaying higher interelectrode standard deviations.

### 3.2. Nonclinical, Maximal Acceptable Dose

None of the three tested Digisonic SP devices reached out-of-bounds measures during the two supplementary fractions of 50 Gy each, although the output current drift continued, with the average output current across the 20 electrodes slowly increasing. The final measurements obtained after a cumulative radiation dose of 160 Gy were +7.73%, SD = 0.37 (SP1); +5.66%, SD = 0.18 (SP2); and +5.90%, SD = 0.35 (SP3). All of the output currents remained within the predefined ±10% acceptable range. Thus, for Digisonic SP devices, we could not determine a maximal acceptable radiation dose below a cumulative dose of 160 Gy.

The three Neuro devices were each subjected to three supplementary sessions of 20, 40, and 150 Gy, delivered over three consecutive days and ultimately pushing the cumulative radiation dose to 290 Gy. Among the three tested devices, only one (Neuro2) broke down at the last session, with the electrodes showing completely erroneous output with a −62.23% deviation from the reference measures (SD = 89.38). The two other devices were still operating inside their specified range after the third supplementary session (290 Gy total), with final deviations of output current measured at −6.52%, SD = 4.43 (Neuro1) and −2.53%, SD = 4.10 (Neuro3). All three devices still showed in-range average output currents after exposure to 100 and 140 Gy. The corresponding values were −3.94% (SD = 2.72) and −5.83% (SD = 3.08), respectively, for Neuro1; −3.81% (SD = 3.62) and −4.93% (SD = 2.09), respectively, for Neuro2; and +1.23% (SD = 1.94) and +1.68% (SD = 4.43), respectively, for Neuro3.

### 3.3. Correlations between Output Current Measures and Cumulative Radiation Dose

To further characterize the relationship between output current measures and cumulative radiation dose, we computed the *R* ^2^ Pearson's correlation coefficients between the 20 electrodes (averaged over the three SP devices) and the cumulative radiation dose. All of the coefficients were significant (Pearson's test, *α* = .05) and ranged between .91 and .95, even when including the two (nonlinear increase) supplementary sessions of 50 Gy. Hence, in Digisonic SP devices, the linear correlation between current outputs and cumulative radiation doses was significant. This relationship was not observed in Neuro devices, for which no reproducible pattern or trend could be observed between devices. In the case of the Neuro devices, none of the 20 correlation coefficients was significant, with values ranging between −0.48 and +0.16 when only including the linear increase period (16 sessions of 5 Gy).

### 3.4. Discussion

The goals of the present study were (1) to test the behavior of two different designs of CI when subjected to a clinical radiotherapy scheme and (2) to determine the maximal acceptable cumulative radiation levels above which the devices would break or show out-of-specification behaviors. A difference in behavior under progressive radiation treatment appeared between the two tested models; in Digisonic SP devices, repeated radiation exposure correlated with a drift of output currents to increased values. This result suggests a cumulative effect of radiation exposure on the devices. This trend was not observed in Neuro devices, for which none of the correlations of the 20 electrodes was significant. This increase in variability, with the nonmonotonicity of the effect of cumulated radiation on Neuro devices compared to Digisonic SP devices, could be a beneficial result of the new design or of the new materials used in this new CI generation that offers better resistance to a cumulative dose of radiation. The observation that there was no device dysfunction at up to 60 or 80 Gy of cumulative radiation is in agreement with previously reported data in the literature. The main data from existing studies on CI irradiation are summarized in Table 1. Ralston and colleagues [6] irradiated six devices (Cochlear Ltd., Sydney, Australia) with 4-MV photons from a linear accelerator (Varian Clinac- 4/80, California) using single fractions of 2 Gy up to a total dose of 50 Gy and then using five fractions of 10 Gy and one fraction of 50 Gy, resulting in a total dose of 150 Gy. Only small changes were reported at the tested irradiation doses: a gradual loss of RF-lock between 50 and 150 Gy and changes in output current at 100 Gy and 150 Gy. Results regarding RF-lock were also reported in a second study of four Clarion 1.2 (Advanced Bionics, California) CI devices. RF-lock was lost for cumulative doses below 50 Gy and for single doses of 20 and 30 Gy. However, these issues were only temporary, as retests 1 min (20 Gy) or 12 h (30 Gy) after irradiation showed that function had returned to normal [5].

Klenzner and colleagues [7] studied the effect of large single doses of radiation on the function of a Nucleus 24k (Cochlear Ltd., Sydney, Australia). Within 2 h, the device was given single fractions of 16.3 Gy, 6.2 Gy, and 20 Gy. No defects were reported in the two measured parameters, namely, impedance and current output, at the total dose of 42.5 Gy. In 2005, Klenzner and colleagues [8] also evaluated the influence of conventional or hyperfractionated radiotherapy on CIs. The conventional fractionation scheme was 2 Gy daily fractions given five times per week, for a cumulative dose of 120 Gy. The hyperfractionation protocol used 1.6 Gy fractions given twice a day on five days per week, for a total of 116 Gy. No dysfunction or permanent failure was observed at total doses of up to 100 Gy for the CIs tested. In only one device, the output current dropped markedly beyond a total dose of 96 Gy. The authors concluded that a total radiation dose of 90 Gy may be considered safe for both implant types tested. One limit of the present research is that the measures were performed only on devices placed onto holders made to accurately represent the conditions of an implant placed onto a patient's head and not on real human patients implanted with a cochlear implant and undergoing real radiotherapy. Of course, given the low number of cases and the high variability of clinical conditions and therapeutic strategies, it remains for the moment difficult to go beyond single-case studies; it would however be interesting to multiply such case reports. To our knowledge, two recent studies included real follow-ups of individual cases of CI patients undergoing radiotherapy [13, 14]. In these two reports, therapeutic schemes employed three doses of 4 Gy (12 Gy) or eight doses of 1.5 Gy (12 Gy). Cumulative doses therefore remained well below those tested in studies not including patients as the present one. Both of these studies reported no dysfunctions in one Combi 40+ (Med-el) and one Nucleus CI512 (Cochlear, Sydney, Australia) devices. Another aspect that could potentially limit the application of radiotherapy in CI patients is that the presence of the metallic implant at the surface of the skull may, at least partially, absorb radiations. This will create a shadowing effect potentially limiting the efficiency of radiotherapy in a relatively large region of the head below the implant. In a former study from our group, we evaluated the absorption caused by the Digisonic SP CI [15]. Results showed that absorption rates were of about 6–7.5%, for a single 6 MV photon beam. This absorption level could be considered as clinically significant for patients with a tumor localized just beneath a cochlear implant. However, radiation treatment planning is usually based on a multibeam geometry and the contribution of beams avoiding the implant could in theory compensate for the attenuation. Further work will be dedicated to extending the present studies to different clinically relevant radiotherapy schemes and to different measures of CI functioning at different stages of the system (RF-Lock, current output, and postradiation exposure quality of the electrical stimulation).

## 4. Conclusions

In the present study, we showed that the Digisonic SP CI devices were not deleteriously affected by radiation up to a total dose of 160 Gy, even when applying single 50 Gy doses. In Neuro CI devices, no out-of-bounds measures were reported up to a total dose of 140 Gy. As changes in the stimulator output current can be compensated simply by reprogramming the processor of the CI, fractionated or hyperfractionated radiotherapy is not contraindicated in patients equipped with CI.

## Figures and Tables

**Table 1:** Review of the scientific literature on cochlear implants and radiation therapy.

Authors, year	Cochlear implants tested (Nb tested)	Implant preparation	Irradiation method	Dose and fractionation scheme (total dose)	Measured parameter, observed dysfunctions, and critical dose
Ralston et al., 1999 [6]	22 MCI (two processor generations) 24MCI (3p./model)	Two water equivalent plastic blocks (30 × 30 × 5 cm), separated by 1 cm thick Perspex blocks	Two parallel opposed 4 MV photon beams	25 × 2 Gy, followed by 5 × 10 Gy and one fraction of 50 (150 Gy)	RF-lock and current output. 50> dose <150 Gy: gradual loss of RF link (CI22M). CI24M changes in output current large at 150 Gy.

Baumann et al., 1999 [5]	Clarion 1.2 (4p.)	10 cm synthetic block, covered by 0.5 cm thick Plexiglas	Cobalt radiation machine, gamma rays @ 1.2 MV	CI1: incr. sequential to (69 Gy) CI2: 3 × 30 Gy (90 Gy) CI3: 34 × 2 Gy (68 Gy) CI4: 29 × 2 Gy (58 Gy)	RF-Lock (communication between ICS and speech processor) loss for dose <60 Gy.

Klenzner et al., 2004 [7]	Nucleus 24 K (1p.)	In situ implantation, cadaver head	Two parallel opposed 6 MV photon beams	16.3, 6.2, and 20 Gy (42.5 Gy)	Impedance and current output. No deficit at tested dose.

Klenzner et al., 2005 [8]	Nucleus CI24M (2p.) Nucleus I24R (2p.)	Solid-water model simulating head tissue, serving as a phantom implanted device	Two parallel opposed 6 MV photon beams	Plan A: 50 × 2 Gy, followed by four fractions of 5 Gy. (120 Gy) Plan B: 60 times 1.6 Gy, followed by four fractions of 5 Gy (116 Gy)	Impedance, current output, and charge balance of biphasic pulse. No dysfunction <80 Gy. Impedance fail at 111 Gy.

Markiewicz et al., 2006 [13]	Combi 40+ (1p.)	Single in situ case study	Total body irradiation, 6 MV photons	3 × 4 Gy (12 Gy)	CI not used on TBI days. No reported dysfunctions.

Reddy et al., 2012 [14]	Nucleus CI512 (1p.)	Single case study	6 MV photons	8 × 1.5 Gy (12 Gy)	No reported dysfunctions.

Current study	Digisonic SP (3p.) Neuro CI (3p.)	Synthetic block, covered by 0.5 cm thick Plexiglas	6 MV photons	12 × 5 Gy (60 Gy) followed by two fractions of 50 (160 Gy) 16 × 5 Gy (80 Gy) followed by 20, 40, and 150 (total 290 Gy)	No more than 10% deviation in output currents at 60 or 80 Gy. One device out of six dysfunctional after a 290 Gy cumulative dose.
